# A Novel Surface-Exposed Polypeptide Is Successfully Employed as a Target for Developing a Prototype One-Step Immunochromatographic Strip for Specific and Sensitive Direct Detection of *Staphylococcus aureus* Causing Neonatal Sepsis

**DOI:** 10.3390/biom10111580

**Published:** 2020-11-20

**Authors:** Sally A. Mohamed, Tamer M. Samir, Omneya M. Helmy, Noha M. Elhosseiny, Aliaa A. Ali, Amani A. El-Kholy, Ahmed S. Attia

**Affiliations:** 1 Department of Microbiology and Immunology, Faculty of Pharmacy, Cairo University, Cairo 11562, Egypt; sally.mohamed@pharma.cu.edu.eg (S.A.M.); omnia.helmy@pharma.cu.edu.eg (O.M.H.); noha.elhuseiny@pharma.cu.edu.eg (N.M.E.); 2 Department of Microbiology and Immunology, College of Pharmaceutical Sciences and Drug Manufacturing, Misr University for Science and Technology, 6th of October City 12566, Egypt; tamer.mohamed@must.edu.eg; 3 Department of Pediatrics, Faculty of Medicine, Cairo University, Cairo 11562, Egypt; draliaaadel@yahoo.com; 4 Department of Clinical Pathology, Faculty of Medicine, Cairo University, Cairo 11562, Egypt; aaakholy@gmail.com

**Keywords:** *Staphylococcus aureus*, neonatal sepsis, direct detection, immunochromatographic strip, cell wall protein, one–step, bioinformatics, rapid

## Abstract

Neonatal sepsis is a life-threatening condition and *Staphylococcus aureus* is one of its major causes. However, to date, no rapid and sensitive diagnostic tool has been developed for its direct detection. Bioinformatics analyses identified a surface-exposed 112-amino acid polypeptide of the cell wall protein NWMN_1649, a surface protein involved in cell aggregation and biofilm formation, as being a species-specific and highly conserved moiety. The polypeptide was cloned, purified, and used to immunize mice to raise specific immunoglobulins. The purified antibodies were conjugated to gold nano-particles and used to assemble an immunochromatographic strip (ICS). The developed prototype ICS detected as low as 5 µg purified polypeptide and 10^2^ CFU/mL *S. aureus* within 15 min. The strip showed superior ability to directly detect *S. aureus* in neonatal sepsis blood specimens without prior sample processing. Moreover, it showed no cross-reaction in specimens infected with two other major causes of neonatal sepsis; coagulase-negative staphylococci and *Klebsiella pneumoniae*. The selected NWMN_1649-derived polypeptide demonstrates success as a promising biomolecule upon which a prototype ICS has been developed. This ICS provides a rapid, direct, sensitive, and specific option for the detection of *S. aureus* causing neonatal sepsis. Such a tool is urgently needed especially in resources-limited countries.

## 1. Introduction

Neonatal sepsis is a life-threatening condition that increases morbidity and mortality rates in newborns especially in developing countries [1]. The World Health Organization (WHO) reported in 2014 that almost 15% of neonatal deaths results from neonatal sepsis especially in low and middle income countries (LMICs) [2]. A traditional reliable technique for the diagnosis of this condition is blood culturing which could require up to seven days [3]. Real-time PCR is a more rapid, reliable, and alternative technique; however it needs well-trained personnel and higher costs [4].

Membrane-based immunochromatographic strips (ICS) are well known and have many advantages such as the low-cost and being a rapid and simple detection method [5]. They also depend on specific antibody-antigen binding [6] and they have been widely used for detecting several pathogens [7,8].

Neonatal sepsis is caused by various microorganisms [2,9]. Data obtained from some neonatal intensive care units in Egyptian hospitals identified *Staphylococcus aureus* as one of the leading Gram-positive neonatal sepsis causes [10,11,12]. Preliminary data, obtained from internal surveillance studies conducted by the Infection Control Unit in Cairo University Hospitals, indicated that the most common causes of neonatal sepsis in these settings were; *Klebsiella pneumoniae, Enterobacter cloacae*, and *S. aureus*. In addition, *S. aureus* is at the top of the list of neonatal sepsis causing pathogens in many countries such as India, Ethiopia, and China [13,14,15].

In this study, we aim to develop a prototype tool to specifically identify *S. aureus* as a causative agent of neonatal sepsis with high degree of sensitivity and in a rapid and simple way. The rapid identification of *S. aureus* would allow the prompt selection of the appropriate antimicrobial therapy, which should contribute to the rapid recovery and improved prognosis.

## 2. Materials and Methods

### 2.1. Statement of Ethical Approval

The protocol of the study was approved by the Research Ethics Committee at the Faculty of Pharmacy, Cairo University (Approval Numbers: MI 1612 and 2426).

### 2.2. Bacterial Strains and Culture Conditions

*S. aureus* strain Newman [16] was used as a reference strain. Cells were grown aerobically in Tryptic Soya Broth (TSB) (Oxoid, Hampshire, UK) at 37 °C. When needed, *S. aureus* was grown on Tryptic Soya Agar (Oxoid, Hampshire, UK) at 37 °C. *Escherichia coli* strains DH5α [17] and BL21 (DE3) [18] were grown aerobically in Luria Bertani (LB) broth (LabM, Lancashire, UK), or on LB agar (LabM, Lancashire, UK) at 37 °C. When appropriate, LB was supplemented with ampicillin (Epico, Tenth of Ramadan, Egypt) to a final concentration of 100 μg/mL. For blue/white colony screening, 5-Bromo-4-chloro-3-indolyl β-D-galactopyranoside (X-Gal) (SERVA, Heidelberg, Germany) was added to a final concentration of 40 μg/mL.

### 2.3. Screening for Potential Targets for Development of Immunochromatographic Strip

#### 2.3.1. Bioinformatics Analyses of the S. aureus Proteome to Identify Potential Targets

The PSORTdb database, v. 3.0 [19] was used to predict the localization of all proteins encoded by *S. aureus* Newman strain genome (*Staphylococcus aureus subsp. aureus* str. Newman DNA, complete genome. NC_009641.1). Proteins predicted to be localized in the cell wall were screened for *S. aureus*-specific proteins or peptides using the BlastP tool [20] against the non-redundant protein database excluding *S. aureus* proteins from query. Proteins that returned no hits or low query coverage were considered to be potentially *S. aureus*-specific. Amino acid (aa) sequences were retrieved and compared by multiple sequence alignment using Clustal Omega, v 1.2.4 [21] to identify highly conserved *S. aureus* regions. Phylogenetic analysis was performed using Unipro UGENE v. 35 platform [22]. The sequences of the potential targets were screened for conserved domains using the NCBI Conserved Domain Database [23].

Physicochemical parameters of the selected potential target were predicted using Expasy’s ProtParam server [24]. Surface exposure of the target peptide was predicted by comparative modeling of the target peptide using Modeller [25] and UCSF-Chimera v. 1.14 [26]. Signal peptide position and cleavage site were predicted using SignalP 4.1 Server which employs neural networks and Markov Models (HMM) to predict signal peptide cleavage site [27]. Subcellular localization of the target peptide was predicted using CELLO online tool v. 2.5 [28]. Probable extracellular domains and transmembrane helices were identified using protein topology prediction tools TOPCONS and CCTOP server version s. 1.00 (Consensus Constrained TOPology prediction web server) [29]; to determine protein regions expected to be exposed on bacterial surface. Molecular weight prediction was performed using the Expasy bioinformatic tool [30].

#### 2.3.2. In Vitro Assessment of the Expression of the Selected Protein Using Real-Time RT-PCR

*S. aureus* Newman cells grown to mid-logarithmic phase were mixed with either phosphate buffered saline (PBS) or heat-inactivated human serum (obtained from healthy volunteers) to a final concentration of 10% (*v*/*v*), and were incubated for two hours at 37 °C. RNA was extracted using the RNeasy minikit (Qiagen, Hilden, Germany) and reverse-transcribed using the QuantiTect reverse transcription kit (Qiagen, Hilden, Germany). The produced cDNA was used as template for real-time PCR analysis using the GoTaq^®^ qPCR SYBR Green master mix (Promega, Madison, WI, USA). Primer pair 16S-Fw (5′-TGAGATGTTGGGTTAAGTCCCGCA-3′) and 16S-Rv (5′-CGGTTTCGCTGCCCTTTGTATTGT-3ʹ) was used for the 16S rRNA normalizer; and primer pair SA029 (5ʹ-CGACTGATGATGGCGTTACT-3′) and SA030 (5′-TCCCTTTGTTTCGTTGCTTTATC-3′) for the *NWMN_1649*. To ensure absence of genomic DNA contamination in the extracted RNA samples, negative RT control sample was tested.

### 2.4. Cloning, Expression and Purification of Recombinant NWMN_1649 Polypeptide

Primers AA726 (5′-GATCATATGAGCGATACTAATCAAGCAACA-3′, NdeI underlined) and AA727 (5′-*TCA*TCCTCCTGAACCATTTTCATTTG-3′, stop codon italicized) were used to amplify the region from nucleotide 136 to 471 of *NWMN_1649* open reading frame (ORF). Primer pair AA667 (5′-CTCCCTTATGCGACTCCTGC-3′) and AA666 (5′-GCCAACTCAGCTTCCTTTCG-3′) was used to amplify a 490 bp fragment including the T7 promoter/His-tag/multi-cloning site (including NdeI restriction site) of the pET15b plasmid (Novagen, Madison, WI, USA). Both fragments were NdeI digested, gel-purified, and ligated. The ligation reaction was used as template in another PCR cycle using primer pair AA667/AA727. The PCR product was then ligated into the rapid cloning vector pLUG-Prime^®^-TA-Cloning Vector II (iNtRON Biotechnology, Gyeonggi-do, Korea) and transformed into DH5α cells. Screening for the right clones was performed using primer pair AA437 (5′-CAGGAAACAGCTATGACCATGATT-3′) and AA438 (5′-GTAAAACGACGGCCAGTGAATT-3′) [31]. The resultant plasmid was designated pNWMN_1649-6xHis and the insert sequence was verified by DNA sequencing. Later, pNWMN_1649-6xHis was transformed into BL21 (DE3) for protein expression.

To purify the polypeptide, overnight culture of BL21/pNWMN_1649-6xHis was diluted in fresh LB/Amp and allowed to grow to mid-logarithmic stage then induced with isopropyl β-D-1-thiogalactopyranoside (IPTG) to final concentration of 1 mM for 16 h at 30 °C. Cells were collected by centrifugation at 4200× *g* at 4 °C, disrupted by sonication till clear solution was obtained; followed by centrifugation at 12,000× *g* at 4 °C. The clear supernatant was then purified using Ni-NTA resin (Qiagen, Hilden, Germany) as described earlier [32]. The polypeptide was analyzed using SDS-PAGE and its concentration was determined using QuantiPro BCA assay kit (Sigma-Aldrich, St. Louis, MI, USA).

### 2.5. Development of an Immunochromatographic Strip(ICS)

#### 2.5.1. Animal Immunization and Production of Immunoglobulins

Ten female BALB/c mice (16–18 g) (Theodor Bilhariz Research Institute, Giza, Egypt) were immunized with 25 µg polypeptide emulsified with Complete Freund’s adjuvant (Sigma-Aldrich, St. Louis, MI, USA) subcutaneously. The mice were boosted after 21 days with 12.5 µg polypeptide emulsified with Incomplete Freund’s adjuvant (Sigma-Aldrich, St. Louis, MI, USA) via the same route then boosted one more time with 12.5 µg polypeptide in PBS intraperitoneally after 2 additional weeks. On day 45, mice were sacrificed, blood was collected by exsanguination of the posterior vena cava, the serum was separated and stored at −20 °C. Mice injected with pyrogen-free saline instead of the polypeptide were used as a negative control.

#### 2.5.2. Specificity Evaluation of the Produced Antibodies using Enzyme Linked Immunosorbent Assay (ELISA)

Immunoglobulins in the immunized mice sera were screened with ELISA using the purified polypeptide. Then the antibodies were purified using the protein A agarose purification kit (KPL, Gaithersburg, MD, USA) according to the manufacturer’s instructions. The antibody titer and specificity against the specific antigen (either in sera or purified immunoglobulins) were assessed with ELISA using 1 μg/well purified NWMN_1649 polypeptide suspended in 50 μL 0.1M carbonate buffer (0.0125M NaHCO_3_ and 0.0875M Na_2_CO_3_, pH 9.5) as the coating antigen; as previously described [33].

#### 2.5.3. Synthesis and Characterization of Gold Nanoparticles (GNPs) 

GNPs were synthesized using the citrate reduction method [34]. The mean particle size of nanoparticles was determined by Photon Correlation Spectroscopy using Malvern Zetasizer Nano-ZS (Malvern Instruments, Malvern, UK).

#### 2.5.4. Preparation of GNP-Antibody Conjugate and Assembly of ICS

The GNP-antibodies conjugate was prepared using the method described by Bailes and co-workers [35] with slight modifications. The stabilization step was done by the addition of 1% BSA (in 20 mM borax buffer, pH 9.2) at ratio of (1:10 *v*/*v*) and incubation for 1 h at room temperature, the mixture was then centrifuged at 12,000× *g* at 4 °C and re-suspended in 2 mM borax buffer (pH 9.2) containing 1% BSA.

ICS preparation and assembly was also performed according to Bailes and co-workers with slight modifications as the glass fiber strip (EMD Millipore, Burlington, MA, USA) (5 mm × 10 mm) was first soaked in the release agent (0.5% Tween-20, 0.5% human serum albumin in water) for 5 min and dried at 37 °C for 2 h. The Ig-gold conjugate was mixed with dispensing agent (20 mM PBS, 5% methanol and 0.1% lactose) at ratio of (4:1 *v*/*v*), loaded onto the dried, treated glass fiber strip and dried at 37 °C for 2 h. The capture antibody was the purified antibodies at concentration of 0.5 mg/mL and the control antibody was 0.5 mg/mL anti-mouse IgG antibody in 0.135 M sodium chloride, diluted (1:5) in dispensing solution to a final concentration of 100 µg/mL, then the procedures were continued as described [35]. The cellulose fiber sample pad (EMD Millipore, Burlington, MA, USA) (15 mm × 5 mm), the treated glass fiber strip, the treated nitrocellulose membrane strip (Carl Roth, Karlsruhe, Germany) (5 × 35 mm) and the absorbent pad were then all assembled.

#### 2.5.5. Testing the Sensitivity and Specificity of the Constructed ICS

The ICS was tested with different amounts of the purified NWMN_1649 polypeptide in 150 µL-aliquots of (10, 5, 2.5, 1.25 and 0.625 µg) and applied onto the sample pad, allowed to flow, and left for 10–15 min before reading the test result. Appearance of two bands at capture and control lines indicated a positive result, while appearance of one band at control line indicated a negative result. Sterile PBS was used as a negative control. To assess the ability of the developed ICS to detect whole *S. aureus* cells, it was challenged with cell suspensions containing different cell concentrations of *S. aureus* Newman (10^2^–10^8^ CFU/mL PBS).

### 2.6. Assessment of the Developed ICS Using Clinical Specimens Obtained from Neonatal Sepsis Patients

A total of ten blood specimens were collected from neonates diagnosed with neonatal sepsis and the causative agents were identified by blood culture followed by VITEK 2. The samples were diluted 10 folds using sterile PBS and 150 µL of each was then loaded onto the sample pad of the assembled ICS and tested as mentioned above.

## 3. Results

### 3.1. In Silico Identification of Potential Targets for ICS

Analyses of the *S. aureus* strain Newman proteome revealed a total of 2919 proteins distributed as follows: 38 in cell wall, 1359 in cytoplasm, 772 in cytoplasmic membrane, 113 extracellular, and 632 with unknown localization. Cell wall proteins, which are most probably surface-exposed, were chosen for further analyses as potentially good diagnostic targets.

**Figure 1:**
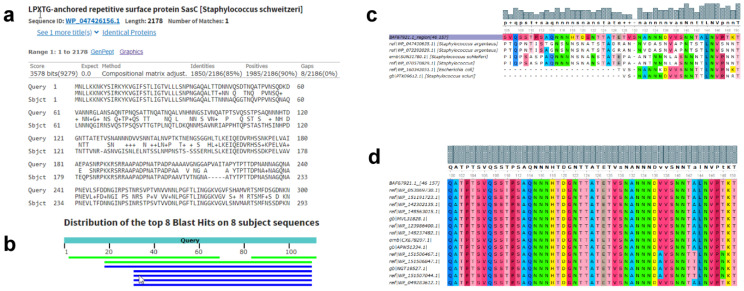
Sequence specificity and conservation of the NWMN_1649 polypeptide. (**a**) Snapshot of the sequence alignment of the first 300 aa of the NWMN_1649 (Query) and its closet matches as determined by the NCBI BLASTp tool. (**b**) Snapshot of the color-coded alignment of the region between aa 46 and 157 of NWMN_1649 and bacterial species other than *S. aureus* as determined by the NCBI BLASTp tool (**c**) Multiple sequence alignment of the target peptide and its closest matches. (**d**) Multiple sequence alignment of the target peptide blast search hits exclusively against multiple *S. aureus* strains. Multiple sequence alignments in (**c**,**d**) were generated using Clustal Omega. The height of the gray bars above the alignment represents the degree of conservation.

Comparative sequence analyses using BlastP tool did not reveal *S. aureus*-specific entire cell wall protein. Accordingly, the search was focused on regions within those proteins that can fulfill the set criteria. Checking the sequence specificity of the first 300 aa of NWMN_1649 (BAF67921.1) of *S. aureus* indicated that the region between aa 46 to 157 looked distinct from the closest match (Figure 1a). Performing a BlastP search using the selected polypeptide against genus *Staphylococcus* while excluding *S. aureus* returned few hits from staphylococcal species not known to cause major human infections, if any, such as *S. schweitzeri, S. argenteus,* and *S. sciuri* (Figure 1b) [36,37]. Multiple sequence alignment using Clustal Omega of the selected peptide and its closest matches from the BlastP analysis (Figure 1c), and phylogenetic tree analysis (Figure S1) confirmed that the *S. aureus* NWMN_1649 (BAF67921.1) 112 aa (46–157) peptide is distinct from its closest matches from other species. Upon, repeating the blast search exclusively against *S. aureus*, the region was 100% conserved in many strains (Figure 1d).

In addition, upon performing a Basic Local Alignment Search Tool (BLAST) analysis using the DNA sequence encoding the current polypeptide (NWMN_1649; aa 46 to 157), it was found to be very highly conserved (% identity 97.917 to 100) in multiple *S. aureus* strains belonging to different sequence types (ST) known to be associated with morbidity and mortality of neonates in neonatal intensive care units, hospital-acquired infections, septicemia and neonatal sepsis such as strains JRA307 (ST 1) [38], JK3137 (ST 8) [39], C8879 (ST 8) [39], M013 (ST 59) [40], CN1 (ST 72) [40], TPS3156 (ST 93) [41], JKD6004 (ST 239) [42], COL (ST 250) [40] as highlighted in Table S1.

**Figure 2:**
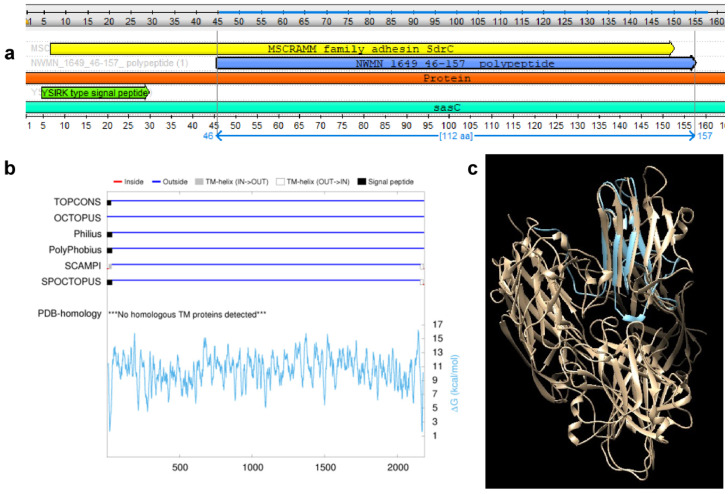
Conserved domains and topology of the NWMN_1649 polypeptide. (**a**) Graphical representation of the first 160 aa from the N-terminal side of NWMN_1649 showing the YSRIK signal peptide domain followed by MSCRAMM family adhesin SdrC domain and the target peptide NWMN_1649 (46–157) is highlighted, the figure was generated from the NCBI conserved domain database. (**b**) Topology prediction of the NWMN_1649 whole protein as determined using the protein topology prediction tools TOPCONS and CCTOP (Consensus Constrained TOPology prediction web server); black boxes indicate the signal peptide, the gray boxes indicate transmembrane helix (in to out), and the blue lines indicate that this sequence is predicted to be extracellular. (**c**) Homology modelling of the NWMN_1649 whole protein with the target peptide (46–157) highlighted in blue being predicted to be surface-exposed and accessible. The modeling was performed using Modeller and UCSF-Chimera.

Searching the NCBI Conserved Domain Database revealed that the parent protein NWMN_1649 (BAF67921.1) (WP_001050548.1) is a bacterial surface protein involved in cell aggregation and biofilm formation [43]. The protein consists of multiple domains, N-terminal side includes the YSIRK type signal peptide and microbial surface components recognizing adhesive matrix molecules (MSCRAMM) family adhesin SdrC domain. The peptide of choice is part of MSCRAMM domain (Figure 2a). The signal peptide cleavage site was predicted by SignalP 4.1 server to be between positions 37 and 38. The target peptide subcellular localization was predicted to be extracellular by CELLO (v2.5).

Protein topology prediction using TOPOCONS confirmed the signal peptide location, while the targeted region is predicated to be extracellular (Figure 2b). Homology model showed that the selected peptide is surface-exposed and accessible (Figure 2c). These analyses indicated that this region could be a potential good target for the proposed diagnostic kit.

### 3.2. Successful Cloning, Expression, and Purification of NWMN_1649 (46–157) Polypeptide

**Figure 3:**
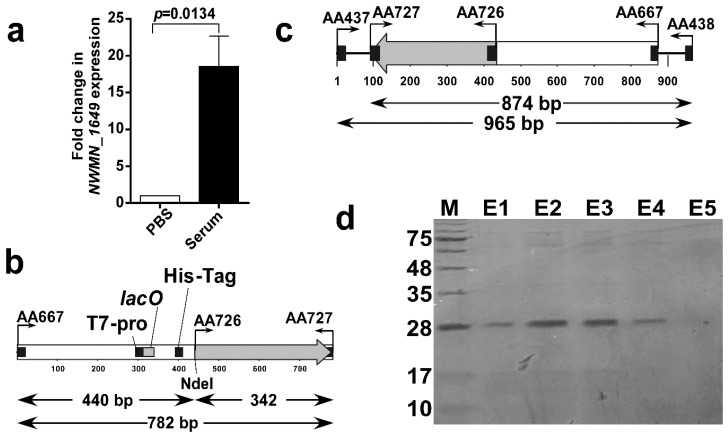
Expression of the NWMN_1649 gene and cloning and expression of the selected polypeptide as a 6xHis fusion. (**a**) Relative expression of the gene encoding NWMN_1649 protein in presence of heat-inactivated human serum. The data presented is the mean of three independent experiments and the error bars represent the standard error. The level of the expression in the presence of PBS was used as a calibrator and the level of expression of the 16S rRNA was used as a normalizer. The statistical significance was determined by the student’s t-test with a *p*-value < 0.05. (**b**) Schematic map of the PCR-ligated fragments, the position of the binding of the respective primers is indicated on the map and the predicted sizes of the respective fragments are indicated below the map. (**c**) Schematic map of the of the multicloning site of pNWMN_1649-6xHis showing the binding sites of different primers and the predicted sizes of the PCR products, the maps in both c and d were generated using the BioEdit Sequence Alignment Editor, v. 7.1.3.0. (**d**) Photograph of 15% SDS-PAGE gel showing the elution fractions “E” of the purification of the cell lysates of the *E. coli* cells carrying the pNWMN_1649-6xHis. The first lane contained a protein marker for molecular mass estimation.

It was crucial to ensure that NWMN_1649 protein is expressed by *S. aureus* under relevant conditions. RT-RT-PCR analyses indicated that NWMN_1649 is expressed either when incubated in PBS or heat-inactivated serum. However, there was a significant expression increase with more than 18-fold (18.51 ± 4.137) when *S. aureus* was incubated in the presence of serum (Figure 3a). Given the results of expression and topology prediction, the NWMN_1649 (46–157) polypeptide is surface-exposed and expressed under normal growth conditions, thus it can be considered a promising diagnostic target.

The DNA fragment encoding the selected peptide was amplified using primer pair AA726/AA727 resulting in the predicted DNA fragment size of 342 bp (Figure 3b and Figure S2A). The DNA fragment of pET-15b vector containing the T7 promotor, the His-Tag, and the multicloning site was amplified with primer pair AA667/AA666 resulting in a 490 bp fragment, upon NdeI digestion of this fragment, it resulted into a 440 bp fragment (Figure 3b). Upon joining the two fragments via DNA ligation followed by PCR amplification with primer pair AA667/AA727, the resultant DNA fragment was obtained with a predicted size of 782 bp (Figure 3b and Figure S2A). The AA667/AA727 fragment was cloned into the rapid TA cloning vector pLUG-Prime^®^-TA-Cloning Vector II and transformed into *E. coli*. Resultant colonies were screened using AA437/AA438 primers which bind on the plasmid backbone outside the cloning site. The clones which gave PCR bands with the predicted fragment size of 965 bp (Figure 3c and Figure S2B) were considered potential right clones. DNA sequencing confirmed the right sequence of the insert and its orientation.

The cloned DNA fragment encoded for a 133 aa polypeptide; 112 aa derived from the NWMN_1649 protein and 21 aa from the fragment cloned from pET-15b including an N-terminal 6xHis-tag fragment. The whole fragment was predicted to be 13.76 kDa with the 112 aa of NWMN_1649 corresponding to 11.45 kDa and the 21 aa from pET-15b correspond to 2.31 kDa (Figure S2C). Upon eluting the His-tagged polypeptide from nickel column and running the fraction in SDS-PAGE gel, prominent band showed up around ~28 kDa (Figure 3d). This band most probably corresponds to the dimeric form of the purified polypeptide.

### 3.3. Assessment of the Obtained Anti-Sera and Construction of the ICS

**Figure 4:**
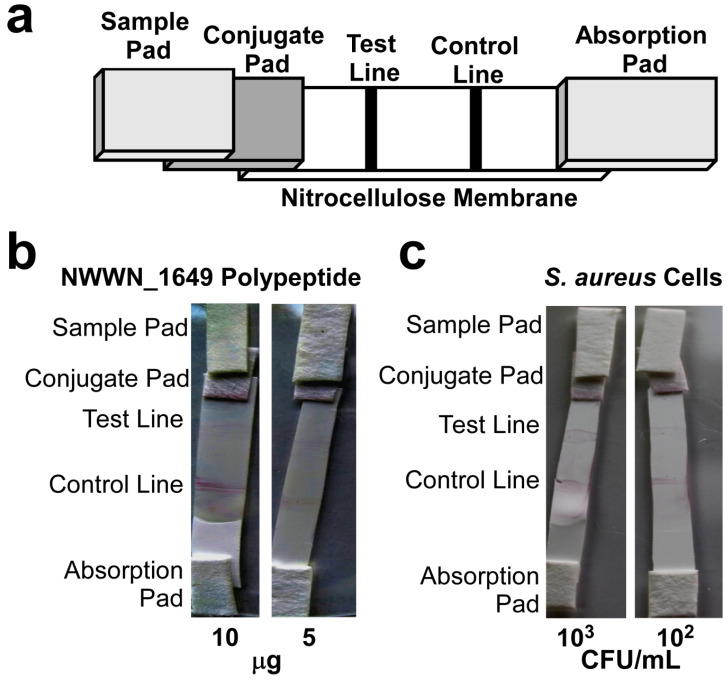
The development of an ICS that can detect both the purified polypeptide and whole cells. (**a**) Schematic diagram showing the design of the developed ICS showing the position and the arrangement of the different components of the strip. (**b**) Photographs of the developed ICS upon testing using different aliquots containing two amounts of the purified polypeptide. (**c**) Photographs of the developed ICS upon testing using aliquots containing two different concentrations of the *S. aureus* cells suspension. In both cases, the sample volume was 150 µL which were loaded on the sample pad and allowed to flow for 10–15 min at room temperature.

The sera of the mice immunized with the NWMN_1649 polypeptide produced titers ranged between 6.4 ×10^4^–2.56 × 10^5^. A diagrammatic sketch of the strip design is presented in Figure 4a. The developed strip was challenged with aliquots of purified polypeptide andwith *S. aureus* cell suspensions. The ICS showed positive signal with as low as 5 µg polypeptide (Figure 4b) and as low as 10^2^ CFU/mL *S. aureus* (Figure 4c).

### 3.4. The Developed ICS Specifically Detects S. aureus in Neonatal Sepsis Samples

**Figure 5:**
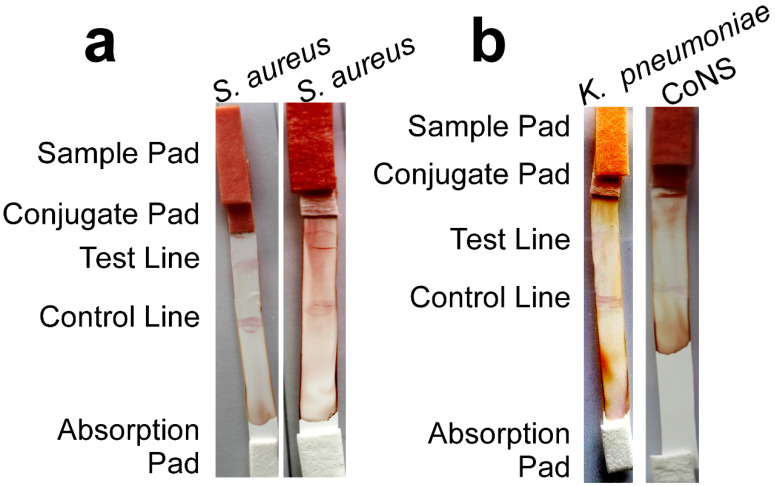
The developed ICS specifically detects *S. aureus* in neonatal sepsis blood samples. (**a**) Photographs of two representative ICS upon testing using two blood samples from neonatal sepsis cases infected by *S. aureus*. (**b**) Photographs of two representative ICS upon testing using two blood samples from neonatal sepsis cases infected by *K. pneumoniae* or coagulase-negative staphylococci (CoNS). One drop (~15 µL) of the blood samples was diluted to 150 µL using sterile PBS prior to loading on the sample pad and allowing it to flow for 10–15 min at room temperature.

Blood was collected from neonatal sepsis cases diagnosed with different microbial infections. The developed ICS gave only positive results with samples infected with *S. aureus* (Figure 5a), while those infected with *K. pneumoniae* or coagulase-negative staphylococci (CoNS) showed negative results (Figure 5b). These results indicate that the developed ICS can specifically detect the target microorganism (*S. aureus*) in this limited pool of real neonatal sepsis samples.

## 4. Discussion

Neonatal sepsis is a leading cause of death in both developed and developing countries [2,44]. Immunochromatographic strip tests are well known for bacterial and viral antigens detection [8,45]. Yet so far, no ICS has been developed to directly detect *S. aureus* in sepsis samples.

Bioinformatics tools have been widely used for drug targets and vaccine candidates’ prediction in various microorganisms [46]. In the current study, an extensive bioinformatics approach managed to identify the target polypeptide. The analyses collectively predicted that this polypeptide is a promising target being species-specific, conserved among many sequenced *S. aureus* strains, and predicted to be surface-exposed, and accessible.

Many *S. aureus* sequence types (ST) have been found in the literature to be commonly associated with morbidity and mortality of neonates in neonatal intensive care units, hospital-acquired infections, septicemia and neonatal sepsis including ST 1, ST 8, ST 59, ST 72, ST 93, ST 239, and ST 250 [47,48,49,50,51,52]. The % identity within the first 500 hits in the BLAST analysis was very high (97.92 to 100%), indicating that this target is highly conserved in *S. aureus* strains in general including those belonging to sequence types known to be involved in fatal infections.

Real-time RT-PCR was used to ensure the optimal expression of the chosen protein candidate under normal growth conditions that would allow the use of this antigen as a target [53]. This tool confirmed the expression of our target protein, NWMN_1649, and its expression up-regulation upon exposure to human serum. On cloning and expression of the selected polypeptide as a 6xHis fusion protein, the produced protein showed up on SDS-PAGE around the size of a dimer rather than a monomeric size. It appeared so with or without using excessive reducing conditions and upon using washing and elution buffers with lower pH and also with high NaCl concentration in washing buffer. This phenomenon has been previously found with several proteins [54,55]. Wu and co-workers showed that the His-tag presence in recombinant purified protein could mediate dimer formation [56]. They suggested that the His-tag could have affinity to an amino acid patch at the protein surface. They gave evidence of His-tag-dependent protein dimerization which should be taken into consideration while analyzing Hig-tagged proteins biochemical properties [56]. Another study has purified recombinant YukE protein, which is a secreted protein of the WXG100 superfamily in *Bacillus subtilis,* and by size exclusion chromatography they showed that it forms a dimer in vitro; the dimer band remained upon changing pH and the monomer was not observed with changing conditions [57]. ELISA has been widely used in previous studies for evaluation of binding of the produced antibodies to their specified antigens [45,58,59]. In this study, we have assessed the specificity of the produced antibodies in the sera of immunized mice with ELISA using purified specific NWMN_1649 polypeptide; which showed good titers.

Several approaches have been employed before for the detection of *S. aureus*. For instance, PCR was used to amplify the *nuc* gene that encodes a thermostable nuclease as a rapid diagnostic tool for *S. aureus* infections by direct testing of the clinical samples. However, performing PCR would require specific equipment and well-trained personnel [60]. Another group developed a method depending on measuring the resonance of the light-scattering signal of aptamer-conjugated-gold nanoparticles; it is low-cost and highly sensitive but it needs a relatively long time (1.5 h) and requires well-trained personnel [61]. Latex agglutination assays were widely used for the detection of *S. aureus* infections as rapid, simple, and sensitive methods; however, they suffered from low specificity especially in the presence of *S. saprophyticus* [62].

A very recent study reported the development of an ICS for detecting *S. aureus* in milk samples [63]. However, they used a ribosomal protein as a target. Accordingly, the detection required excessive sample processing including cell wall digestion and cell lysis prior to application on ICS which is a major drawback that has been avoided in the current study. Moreover, their limit of detection was as high as 10^4^ CFU/mL. In addition, in 2014, Niu and co-workers have developed an ICS to detect *S. aureus* in simulated food samples and had a limit of detection as low as 10^3^ CFU/mL [64]. This ICS was based on a commercially available monoclonal antibody (ab37644)that cross-reacts with CoNS *S. epidermidis* according to the vendor’s description (Abcam, Cambridge, MA, USA)and this mAb production has been discontinued in June 2020. In addition, other studies were directed toward *S. aureus* detection in food specimens. However, they also required extensive sample treatment before testing [65,66]. This was avoided in the current study and we could directly test blood samples without prior treatment which would save time and effort. The designed strip had reaction time of 10–15 min which is similar to those described in previous studies [7,67]. Another study designed ICS for *S. aureus* detection in respiratory samples [58]; although direct sample detection was possible, the detection limit was too high 10^6^ CFU/mL and the reaction toward *S. epidermidis* was not evaluated, which is a major blood contaminant in neonatal sepsis and can easily cross-react with antibodies to *S. aureus* antigens. It is worth mentioning that the ICS developed in the current work was non-reactive to a cell suspension of *S. epidermidis*, which is an additional advantage to the current tool. Accordingly, an additional advantage of the current ICS is its low limit of detection, as low as 10^2^ CFU/mL of *S. aureus*, which is at least 10-fold less than previously reported systems.

Potential limitations of the current study may include the lack of sensitivity of the developed ICS to some *S. aureus* strains belonging to sequence types which were not represented in the 500 hits checked in the BLAST analysis. This could be possibly addressed in future studies by expanding the range of the isolates tested to ensure good coverage of all the possible sequence types.

## 5. Conclusions

In this study, we have applied an innovative bioinformatics approach to identify a specific, surface-exposed biomolecule to be used as a successful target for the development of a sensitive, specific, simple, and rapid ICS for detecting *S. aureus* causing neonatal sepsis. The ICS successfully provides a promising alternative prototype for *S. aureus* detection especially in resources-limited countries. Future plans include large scale clinical testing of the developed prototype and usage of the selected antigen to generate specific monoclonal antibodies to maintain a sustainable source for production of this ICS.

## Supplementary Materials

The following are available online at https://www.mdpi.com/2218-273X/10/11/1580/s1, Figure S1: Phylogenic tree of the blast data in tree representation showing target peptide relationship to its nearest blast hits. Figure S2: Confirmation of the NWMN_1649-6xHis fusion protein construct. Table S1: Results of BLAST analysis of the DNA sequence encoding the target peptide with results of the strains belonging to different STs known to be involved in neonatal infections highlighted.
