# Supplementary material for: A Novel Surface-Exposed Polypeptide Is Successfully Employed as a Target for Developing a Prototype One-Step Immunochromatographic Strip for Specific and Sensitive Direct Detection of Staphylococcus aureus Causing Neonatal Sepsis
Source: Biomolecules. 2020 Nov 20;10(11):1580. doi: 10.3390/biom10111580 (PMC7699858; doi:10.3390/biom10111580)
Supplement: Supplementary file 1 [file biomolecules-10-01580-s001.zip › Mohamed_et_al_2020_Figure_S1.docx]

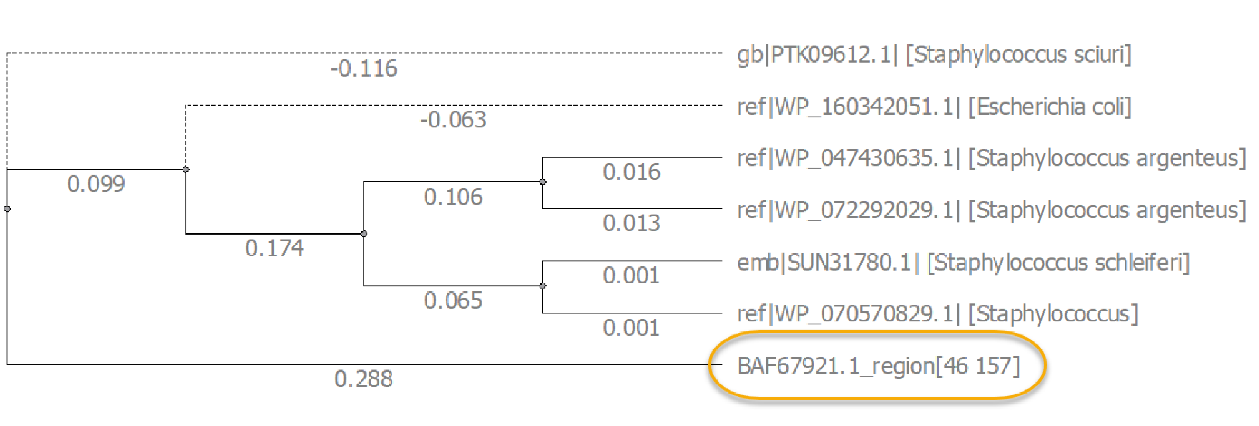


**Figure S1.** Phylogenic tree of the blast data in tree representation showing target peptide relationship to its nearest blast hits. The tree was generated using the Unipro UGENE v. 35 bioinformatics tool.
