# Supplementary material for: A Novel Surface-Exposed Polypeptide Is Successfully Employed as a Target for Developing a Prototype One-Step Immunochromatographic Strip for Specific and Sensitive Direct Detection of Staphylococcus aureus Causing Neonatal Sepsis
Source: Biomolecules. 2020 Nov 20;10(11):1580. doi: 10.3390/biom10111580 (PMC7699858; doi:10.3390/biom10111580)
Supplement: Supplementary file 1 [file biomolecules-10-01580-s001.zip › Mohamed_et_al_2020_Figure_S2.docx]

**Figure S2. Confirmation of the NWMN_1649-6xHis fusion protein construct.** (**A**) Photograph of an agarose gel showing the sizes of the AA726/727 and AA667/AA727 PCR fragments showing up at their predicted sizes. (**B**) Photograph of an agarose gel showing the PCR product generated using primer pair AA437/AA438 confirming its predicted size. The first lane of each gel contained a DNA bp ladder for size estimation. (**C**) Schematic diagram of the constructed polypeptide showing the predicted sizes of each fragment and the whole polypeptide.
